# Effect of spinal manipulative treatment on cardiovascular autonomic control in patients with acute low back pain

**DOI:** 10.1186/s12998-017-0167-6

**Published:** 2017-12-04

**Authors:** Mohamed Younes, Karine Nowakowski, Benoit Didier-Laurent, Michel Gombert, François Cottin

**Affiliations:** 1 CIAMS, Université Paris Sud, Université Paris-Saclay, F-91405 Orsay, France; 2 CIAMS, Université d’Orléans, F-45067 Orléans, France; 3 ESO Paris SUPOSTEO, F77420 Champs-sur-Marne, France

**Keywords:** Baroreflex, Blood pressure, Heart rate, Autonomic nervous system, Back pain, Spinal manipulation, Randomized study, Pain scale

## Abstract

**Background:**

This study aimed to quantify the effect of spinal manipulative treatment (SMT) from an analysis of baroreflex, systolic blood pressure and heart rate variability (HRV) on patients with acute back pain. It was hypothesized that SMT would increase the parasympathetic cardiovascular autonomic control.

**Methods:**

Twenty-two patients with acute back pain were randomly divided into two groups: one receiving sham treatment (Sham) and the other receiving SMT. Recordings were completed during the first day and the seventh day, immediately before and after treatment on both days. ECG and systolic blood pressure were continuously recorded to compute cardiovascular variability and baroreflex sensitivity components. The perceived level of pain was measured with the numeric pain scale (NPS) 48 h before, just before and just after each treatment. The NPS ranged from 0 to 100% (peak of pain before treatment). ECG and systolic blood pressure recordings were analyzed in time frequency domain using the Smoothed pseudo Wigner-Ville distribution.

**Results:**

Root mean square of the successive differences, high frequency power of the heart rate variability, and high frequency baroreflex sensitivity differences between post and pre tests were higher in the SMT group than in the Sham group (*p* < 0.01), whereas no differences were observed with the other heart rate variability components. Also, no differences were observed with the systolic blood pressure components. Although the estimated pain scale values decreased over time, no difference was observed between the SMT and Sham groups.

**Conclusions:**

This seems to be the first study to assess the effect of SMT on both heart rate variability and baroreflex sensitivity in patients with acute back pain. SMT can be seen to provoke an increase in parasympathetic control known to relate to a person’s healthy state. Thus, cardiovascular variability analysis may be a useful tool for clinicians to quantify and objectify the beneficial effects of spinal manipulation treatment.

## Background

Nearly two thirds of adults are affected by low back pain at some point in their lives^.^ [1]; indeed, it accounts for 97% of all pathologies arising from spinal structures^.^ [2]. Low back pain involves the occurrence of pain in the space between the rib cage and gluteal fold and can also be defined as acute, recurrent, or chronic episodes [3]. Low back pain symptoms can derive from many anatomic sources, such as nerve roots, muscles, fascial structures, bones, joints, intervertebral discs, and organs within the abdominal cavity [4]. Spinal manipulative treatment (SMT) as a treatment for mechanical pain has been practiced for centuries. The rationale for this treatment is, for example, that it may decrease the sensitivity of the muscle spindles and/or the various segmental sites of a reflex pathway [5]. SMT is also thought to affect reflex neural outputs to both muscle and visceral organs by affecting both paraspinal muscle reflexes and motor neuron excitability [6]. In the literature, several studies have identified the effect of SMT on the heart rate variability (HRV)^.^ [7, 8]. To our knowledge, the effect of SMT on both HRV and baroreflex sensitivity has not previously been studied. This is relevant, as they may be relevant additional indices in quantifying the physiologic effects of SMT.

The HRV and baroreflex sensitivity can be analyzed in such a way as to provide several indices of cardiovascular autonomic control [9]. Signal processing of successive inter-beat intervals (RR) from electrocardiogram (ECG) and systolic blood pressure (BP) allows the quantification of the sympathovagal balance, which is believed to reflect the cardiovascular health of humans at rest^.^ [10]. In a nutshell, pathological and stress states shift the sympathovagal balance towards an increase in sympathetic cardiac control^.^ [10], whereas healthy cardiovascular states shift the sympathovagal balance towards an increase in parasympathetic cardiac control^.^ [11]. Furthermore, a decrease in baroreflex sensitivity has been associated with pathological states such as chronic heart failure^.^ [12], hypertension^.^ [13], and other human pathophysiological states [12, 13].

To quantify these short term modulations of BP and HR, spectral analysis has been used [14, 15] providing two main frequency components: a low frequency (LF) ranging from 0.04 to 0.15 Hz [10] and a high frequency (HF) centered at the breathing frequency [10]. At rest, the quantification of spectral components give indices of the autonomic control of HR and BP. On the one hand, it has been shown that the HF spectral component of HR variability (HF-HRV) is an index of the vagal tone [10, 16], whereas both sympathetic and vagal activities contributed to LF (LF-HRV) spectral component of HRV [10, 16]. On the other hand, LF spectral component of systolic BP variability (SBPV) only reflects the sympathetic activity to the alpha-adrenergic receptors of vasculature [17] whereas HF-SBPV probably reflects the mechanical effect of breathing on SBP [9, 18].

Linked to pathological states, it has been shown that pain has an effect on cardiovascular control through an increase in heart rate^.^ [19–21], shown as an increase in the sympathetic indices of cardiac control, such as low frequency (LF) power, and a low to high frequency power ratio (LF/HF) of HRV^.^ [21, 22]. Moreover, nocturnal HRV indices, indicative of sympathetic predominance, were found to be significantly different in women suffering from fibromyalgia as compared to healthy individuals^.^ [23]. In addition, elevated resting blood pressure and spontaneous baroreflex sensitivity appear to be associated with hypoalgesia in acute pain^.^ [24]. Other studies have found a negative correlation between pain threshold and low frequency of the baroreflex sensitivity in patients with hypoalgesia^.^ [22].

Conversely, chiropractic care^.^ [7], cervical adjustments^.^ [25], SMT and a single session of manual therapy program^.^ [26] have been shown to cause pain decrease associated with a shift of the sympathovagal balance toward parsympathetic cardiac control.

Although some studies on the effect of manipulative treatments on HRV have been published^.^ [7, 8], the literature about the relationship between SMT and blood pressure control remains scarce^.^ [27]. However, in addition to HRV, an analysis of baroreflex sensitivity and blood pressure variability can offer valuable information, allowing us to identify additional indices of cardiovascular control to quantify the beneficial effects of SMT. Therefore, such an analysis can provide objective indices that allow us to quantify the effect of SMT on the cardiovascular autonomic control. Thus, we hypothesized that SMT can shift the sympathovagal balance towards an increase in parasympathetic cardiovascular autonomic control. If this were the case, cardiovascular variability could be used to quantify the effects of SMT.

Therefore, the objective of the present study was to quantify the effect of SMT by comparing the results of SMT and sham treatment on (i) the baroreflex sensitivity, (ii) the systolic blood pressure variability, and (iii) the HRV in subjects with acute back pain. In addition, we compared the level of self-reported pain in the two study groups.

## Methods

### Subjects

Twenty-two male volunteers with acute mechanical back pain were enrolled in the study. The patients were randomly divided into two groups: one receiving sham treatment and the other receiving SMT treatment. All patients received exactly the same type and amount of interventions based on the intensity and area of pain. All volunteers were patients attending the Osteopathy Higher School Clinic in Paris and had received manual therapy within the preceding 6 months. The subjects were recruited through personal contact.

Only male patients were included in order to avoid the possibility of menstrual hormone interferences. All were free of cardiac or pulmonary disease, any analgesic treatment, and had not ingested any caffeine or alcohol for 72 h prior to each experimental session. All patients had suffered acute pain for less than 3 months, with a numeric pain scale greater than 5/10 in the last 48 h. The 22 patients were split randomly into two groups by drawing lots. The treating clinician and the person doing the assessment were not involved in the treatment allocation.

### Procedure

This study was carried out in a double blind fashion (i.e. study subjects and person who collected and analyzed the data) and carried out in accordance with The Code of Ethics of the World Medical Association (Declaration of Helsinki). Before measurements were taken, participants were familiarized with the experimental procedure and informed of the risks associated with the protocol. Ethics committee approval (N° 06–0316-CIAMS, UFR STAPS Orsay, France) and written informed consent of the participants was obtained, including the authorization to publish their data.

#### Clinical examinations

Each patient underwent a clinical examination of the thoraco-lumbar spine (including observation, amplitudes of movement, orthopaedic tests, and palpation for pain) by an osteopath to confirm the lumbar origin of the symptoms and to determine the vertebral level at the origin of the pain in sitting position. In addition, lumbar and pelvic muscles were palpated in search of associated myofascial pain. The palpation in search of painful reproduction has been recommended to locate the treatment site in manual therapy [28].

#### Spinal manipulative treatment

The treatment could consist of various techniques of vertebral manipulation (various types of high-velocity, low-amplitude (HVLA) vertebral manipulations or passive mobilization) and muscular manipulations, depending on the tolerance of the patient, as follows:

- The HVLA classical manipulation [29]: the patient was positioned in a side-lying position, the therapist made contact with his hand on the lumbar vertebral level causing the symptoms and applied a tensioning force immediately followed by a rapid impulse in a postero-anterior direction with respect to the patient’s spine.
- Lumbar mobilization: the patient was positioned in a side-lying position or on the stomach depending on the least painful position. The therapist contacted the lumbar vertebral segment in the symptomatic area with one or two hands and applied a rhythmic oscillation without impulse. The mobilization was applied 3 times during 30 s, with a one-minute pause between each application.
- Muscular manipulations: the patient was positioned in a ventral position, Myofascial pain in the lumbar and gluteal region was treated with ischemic pressure [30], deep transverse massage [30], strain counter strain [30], and/or Muscle Energy Technique [30] depending on the patient’s tolerance and whether or not it was possible to perform the technique on the incriminated muscles.

The SMT intervention lasted 45 min and the subjects received exactly the same type and amount of vertebral manipulation by the same person. The protocol was standardized, and was performed by techniques taught at the Higher School of Osteopathy^.^ and referenced in the Glossary of Osteopathic Terminology^.^ [30].

The Sham intervention simulated these techniques, but with improper patient positioning, deliberately misdirected movements, and diminished treatment provider force^.^ [31]. The therapist applied manual contact to the area at the origin of the pain as in. the treatment group but without using impulse or rhythmic movements. For muscle pain, the therapist used a light contact without movement on the myalgic cord.

### Experimental design

**Fig. 1:**
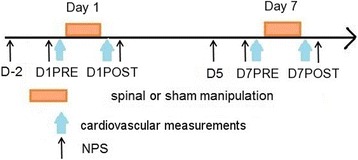
Schematic drawing of the experimental design. NPS refers to the numeric pain scale. D(−2) refers to 2 days before the day of the first treatment (D1), D1PRE to just before the first treatment, D1POST to immediately after the first treatment, D (5) to 5 days after the first treatment, D7PRE to just before the second treatment, and D7POST to immediately after the second treatment

The study took place over 7 days. On day 1 (D1) and day 7 (D7), two recording phases were performed: one before and one after the SMT or Sham interventions, respectively (Fig. 1). All patients received exactly the same type and amount of interventions. Each recording phase and treatment was achieved at the same time of day and under the same conditions (i.e., operator, practitioner, room and equipment used). The mean of difference scores of cardiovascular variables between POST and PRE treatment was used to compare the two groups (SMT vs. Sham). This difference was also compared for the self-reported level of pain.

### Cardiovascular measurements

Before and after SMT or Sham intervention, cardiovascular signals (blood pressure and ECG) were continuously recorded during in total of 35 min. The protocol included four recording phases of 7 min each. The patient’s breathing frequency was paced at 0.25 Hz, imposed by an audio recording feedback. Recordings began 15 min after patients were placed supine in a quiet room at a constant temperature (22 °C) in order to ensure that heart rate level was accommodated to the participant’s posture.

ECG data were recorded and digitized using a PowerLab device (ADInstruments Ltd., AUS) at a sampling frequency of 1000 Hz.

A Finometer device (TNO, BMI, Netherlands) recorded blood pressure, using a cuff placed on the middle finger. The blood pressure signal was displayed by the Finometer device, which had been experimentally validated during previous laboratory tests^.^ [32, 33]. The Finometer was connected to the PowerLab, which digitized and sampled the blood pressure signal at 1000 Hz.

### Pain level measurements

The perceived level of pain was measured with the numeric pain scale (NPS). The NPS is a 100 mm line on which the patient places a single “X” to record the amount of pain perceived^.^ [34, 35]. The NPS is measured as the distance from the left end of the line to the X and is scored out of a possible 100. NPS was administered at baseline and after manipulation had been given. Patients provided their pain scores 48 h before, just before and just after each treatment (Fig. 1). All scores were subsequently normalized, with 100% corresponding to their highest score (peak of pain before treatment). The subsequent scores were calculated as a percentage of their highest score.

### Signal processing

Beat to beat (RR) intervals were extracted from ECG, and systolic BP from blood pressure signals (Chart7 pro, ADInstruments, AUS). Before using the smoothed pseudo Wigner-Ville distribution (SPWVD) processing, occasional ectopic beats (artifacts, cumulative RR and BP periods, and extrasystolic beats/recordings) were identified, visually and manually, and replaced with interpolated adjacent RR and BP interval values using control Excel with macros.

The root mean square differences of successive RR intervals (RMSSD) were calculated as following:

$$ \mathrm{RMSSD}=\sqrt{\frac{1}{N-1}\left[\sum \limits_{i-1}^{N-1}{\left( RRi+1- RRi\right)}^2\right]} $$

Where N = number of R-R interval terms.

All RR series were then re-sampled at 4 Hz, using a cubic spline function.

### The smoothed pseudo Wigner-Ville distribution (SPWVD)

The SPWVD was used to compute instantaneous components of HRV and systolic blood pressure (SBP) variability. It was performed with the cardiovascular toolbox developed in a scientific laboratory, SCILAB (INRIA, France). The SPWVD provides a continuous evaluation of amplitude and frequency, giving a nearly “instantaneous” complex of the fast Fourier transform (FFT) spectrum for each beat, with a high resolution achieved by independent time and frequency smoothing. Then, according to the characteristics of measurement^.^ [10], the instantaneous time frequency components were computed in low (LF-RR and LF-SBP from 0.04 to 0.15 Hz) and high frequency (HF-RR and HF-SBP from 0.15 to 0.4 HZ) bands of SBP variability and HRV, as follows:

$$ LF=\sum \limits_{f=0.04}^{0.15} PDF.\Delta f\; and\; HF=\sum \limits_{f=0.15}^{0.4} PDF.\Delta f\;\left({ms}^2\right) $$

#### Baroreflex sensitivity

The baroreflex sensitivity was assessed in LF and HF bands at rest. The spectral baroreflex sensitivity reflects the linear relation between the input (BP) and output (RR) of the model. The degree of linearity between the two signals was estimated using the value of the coherence function. Ranging from 0 to 1, the RR and SBP spectra were shown to have a reliable linear relationship when the coherence index was higher than 0.5^.^ [36, 37]. The averaged spectral gain in high frequency and low frequency bands was the modulus of the transfer function between the RR and SBP spectra^.^ [12, 38, 39].

### Statistical analysis

All data sets were initially tested for normality using a Kolmogorov-Smirnov test.

In order to determine the effect of treatment (SMT vs. Sham) and time conditions (D1 vs. D7) on cardiovascular variables and NPS score, a two-way analysis of variance (ANOVA) was performed. The mean of difference scores of cardiovascular variables between POST and PRE treatment was used to determine the effect of treatment (SMT vs. Sham).

All analyses were performed with Sigma Stat software (version 3.5, 2007, Systat Software Inc. San Jose, CA, USA). The threshold for statistical significance was set to *p* < 0.05. All data are reported as means and SEM. Whenever the difference was significant, the effect size was computed with the Cohen’s d. It can be calculated as the difference between the means divided by the pooled SD^.^ [40]. Effect size indexes and the conventional values thereof are given for operationally defined small (around 0.2), medium (around 0.5), and large (around 0.8) effects.

## Results

Five patients (3 SMT and 2 Sham) were excluded from the study because they did not participate in the second session. There was no obvious difference between the SMT and Sham groups with regard to age, height and weight (mean, SD).

- SMT intervention, (age: 31 +/− 9 years; height: 178 +/− 8 cm; weight: 75 +/− 12 Kg).
- SHAM intervention, (age: 28 +/− 7 years; height: 176 +/− 7 cm; weight: 70 +/− 15 Kg).

Pretreatment physiologic baseline (D1PRE) heart rate variability, systolic blood pressure variability and baroreflex sensitivity components were not significantly different between each baseline period (*p* > 0.15), as shown in Table 1. In addition, the enforced breathing frequency (*f*HF) remained constant with an instantaneous *f*HF very close to 0.25 Hz during all the measures.

**Table 1:** Average scores of cardiovascular baseline components before treatment measured during the two sessions of treatments between SMT and SHAM

Autonomic cardiac components (*n* = 17)	SMT (*n* = 10)	SHAM (*n* = 7)	Significance
RR period (ms)	826 (47)	847 (43)	0.481
RMSSD (ms)	38.9 (2.18)	39.2 (2.3)	0.931
Log LF-RR (ms^2^)	3.08 (0.06)	3.10 (0.09)	0.652
Log HF-RR (ms^2^)	3.00 (0.04)	3.10 (0.04)	0.151
LF/HF	1.26 (0.15)	1.08 (0.16)	0.429
100.LF/(LF + HF) %	54.3 (2.7)	50.4 (3.5)	0.393
100.HF/(LF + HF) %	46.7 (2.7)	49.6 (3.5)	0.393
SBP (mm Hg)	118 (4)	122 (2)	0.422
LF-SBP (mm^2^Hg)	11.15 (0.75)	12.78 (0.71)	0.151
HF-SBP (mm^2^Hg)	1.44 (0.31)	2.02 (0.39)	0.254
LF-BRS gain	14.20 (1.12)	13.84 (1.05)	0.826
HF-BRS gain	20.21 (2.35)	21.42 (2.10)	0.720

There is no significant difference between groups (SMT vs. SHAM)

*SMT* spinal manipulative treatment, *RMSSD* root mean square of the successive differences, *LF* low frequency, *HF* high frequency, *SBP* systolic blood pressure, *BRS* baroreflex sensitivity. Data are presented as mean (SEM)

### Effects of interventions (SMT vs. Sham)

**Fig. 2:**
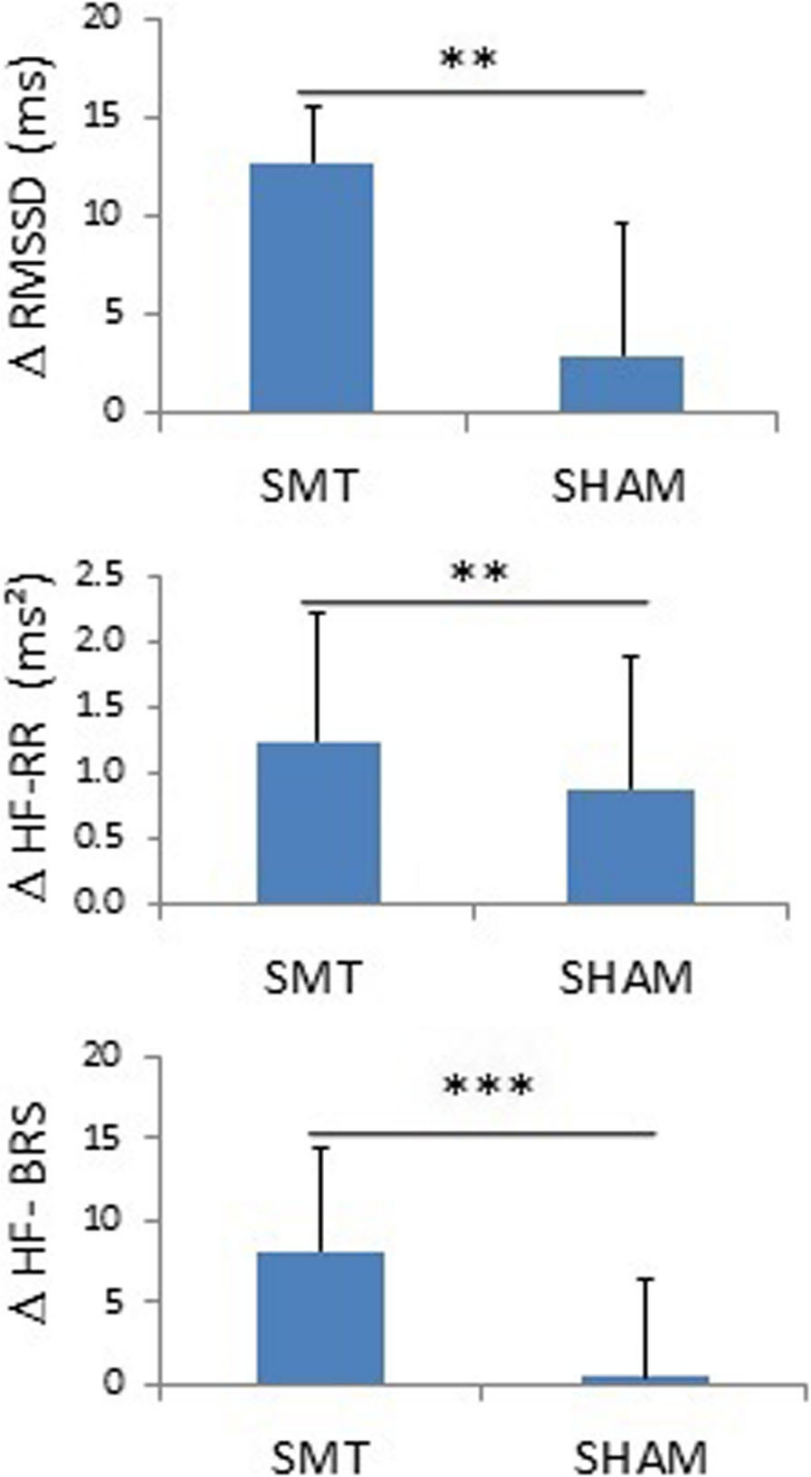
Effects of the treatment (SMT vs. SHAM) on the vagal components of cardiovascular variability analysis based on the mean of difference scores between POST and PRE treatment within groups at the two sessions of treatment. Δ, difference between POST and PRE treatment. SMT refers to spinal manipulative treatment, and RMSSD to root mean square of the successive differences. HF refers to High frequency and BRS refers to baroreflex sensitivity. ***represents *p* < 0.001 and ** represents *p* < 0.01, *n* = 17

Spinal manipulation had an effect on several cardiovascular control indices which is manifested by an increase in HRV indices and vagal modulation (Fig. 2, Table 2). With the HRV indices (RMSSD and HF), which reflect vagal modulation, RMSSD (*p* = 0.003, effect size = 0.52, Table 2) and HF (*p* = 0.005, effect size = 0.33, Table 2) were higher for patients who received SMT than for those undergoing Sham intervention, whereas no difference was observed with all other HRV components (Table 2).

**Table 2:** Effects of treatment (SMT vs. SHAM) and experimental period (D1 vs. D7) on cardiovascular components

Autonomic cardiac components (*n* = 17)	Normality test	Treatment			Period		Significance
		SMT (*n* = 10)	SHAM (*n* = 7)	Significance	D1 (*n* = 17)	D7 (*n* = 17)	
ΔRR period (ms)	Failed	47.14 (19.06)	36.97 (22.78)	0.735	64.34 (21.00)	19.77 (21.00)	0.144
ΔRMSSD (ms)	Passed	12.70 (1.93)	2.85 (2.31)	0.003*	7.94 (2.13)	7.61 (2.13)	0.912
ΔLog LF-RR (ms^2^)	Passed	155.57 (81.49)	−5.26 (97.40)	0.21	25.52 (89.80)	124.79 (89.80)	0.441
ΔLog HF-RR (ms^2^)	Passed	223.08 (74.80)	−125.24 (89.40)	0.006*	109.22 (82.42)	−11.38 (82.42)	0.309
ΔLF/HF	Passed	−0.10 (0.11)	0.14 (0.13)	0.160	−0.07 (0.12)	0.12 (0.12)	0.258
Δ100.LF/(LF + HF) %	Passed	−0.02 (0.02)	0.03 (0.028)	0.155	−0.01 (0.02)	0.02 (0.02)	0.226
Δ100.HF/(LF + HF) %	Passed	0.02 (0.02)	−0.03 (0.028)	0.155	0.01 (0.02)	−0.02 (0.02)	0.226
ΔSBP (mm Hg)	Passed	7.32 (2.87)	3.66 (3.43)	0.420	2.42 (3.16)	8.56 (3.16)	0.180
LF-SBP (mm^2^Hg)	Passed	−0.42 (0.88)	0.03 (1.06)	0.745	−0.58 (0.97)	0.19 (0.97)	0.576
ΔHF-SBP (mm^2^Hg)	Failed	0.759 (0.417)	0.63 (0.49)	0.846	0.73 (0.46)	0.66 (0.46)	0.916
ΔLF-BRS gain	Passed	−0.23 (1.03)	0.22 (1.24)	0.777	−0.94 (1.14)	0.93 (1.14)	0.255
ΔHF-BRS gain	Passed	8.10 (1.45)	0.30 (1.73)	0.002*	4.34 (1.60)	4.06 (1.60)	0.902

Δ, difference between POST and PRE treatment, *SMT* spinal manipulative treatment, *RMSSD* root mean square of the successive differences, *LF* low frequency, *HF* high frequency, *SBP* systolic blood pressure, *BRS* baroreflex sensitivity. Data are presented as mean (SEM). Boldface * represent *p* < 0.05

The baroreflex sensitivity in the HF band (which reflects vagal modulation) differed in the two groups, with a higher gain in the SMT than in the Sham group (Fig. 2, Table 2, *p* < 0.001, effect size = 0.53, whereas no difference was observed with LF.

Neither the SBP nor the SBP variability indices differed between the SMT and Sham groups. With NPS, no effect of the treatment was observed (SMT: 28.01 ± 2.55% vs. Sham: 29.23 ± 3.04%, NS, Table 3).

**Table 3:** Effects of the experimental period on numeric pain scale (NPS) score between and within groups of treatment (SMT vs. HAM)

Experiemental period (*n* = 17)	SMT (*n* = 10)	SHAM (*n* = 7)	Significance between groups
D-2	50.50 (6.25)	42.85 (7.46)	0.43
D1PRE treatment	28.70 (6.24)	29.28 (7.46)	0.95
D1POST treatment	15.30 (6.24)^b^	25.71 (7.46)	0.28
D5	36.60 (6.24)^a^	40.57 (7.46)	0.68
D7PRE treatment	24.40 (6.24)	18.85 (7.46)	0.57
D7POST treatment	12.60 (6.24)^b^	18.14 (7.46)	0.57
All experimental period	28.01 (2.55)	29.23 (3.04)	0.75
Significance within groups	0.001*	0.09	

D-2 refers to 2 days before the day of the first treatment (D1), D1PRE to just before the first treatment, D1POST to immediately after the first treatment, D5 to 5 days after the first treatment, D7PRE to just before the second treatment, and D7POST to immediately after the second treatment. Data are presented as the mean (SEM). Mean values followed by different letters (^a^, ^b^) indicate a significant difference of experimental period within SMT group. Boldface * represent *p* < 0.05

### Effects of the experimental period

**Fig. 3:**
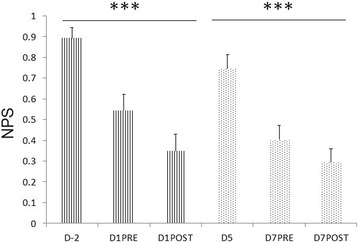
Effects of the experimental period on numeric pain scale (NPS) score including both treatment groups. D-2 refers to 2 days before the day of the first treatment (D1), D1PRE to just before the first treatment, D1POST to immediately after the first treatment, D5 to 5 days after the first treatment, D7PRE to just before the second treatment, and D7POST to immediately after the second treatment. *** represents *p* < 0.001. *n* = 17

No difference was observed in NPS and all the components of cardiovascular variability between the first (D1) and the second session (D7) of treatment for the two groups (Tables 2, 3). With NPS, the score decreased over time during the first and the second session of treatment for both treatment groups (PRE and POST, *p* < 0.001, Fig. 3). However, we did not find a significant difference in NPS score between the two sessions of treatment (Table 3).

## Discussion

### Summary of findings

The main objective of this study was to quantify the SMT effectiveness in patients with acute low back pain from the analysis of cardiovascular variability. The main results of this study were that the components of cardiovascular variability linked to the vagal modulation of heart rate (RMSSD, HF-RR and HF of baroreflex sensitivity) were significantly higher with SMT than Sham intervention (Fig. 2). Indeed, RMSSD, HF power of HRV and HF of baroreflex sensitivity are known to be related to parasympathetic cardiac control^.^ [41, 42]. Thus, when these components are high, this tends to reflect a good sensitivity of the cardiac autonomic control. It seems, therefore, that the results of the baroreflex, heart rate, and SBP variability analysis confirmed the hypothesis that the SMT shifts the sympathovagal balance towards an increase in parasympathetic cardiovascular control. However, no effect was noted on the self-reported level of pain.

### The autonomic nervous system

These findings were consistent with those reported by Giles^.^ [8], who noted an increase in the HF spectral power of HRV linked to SMT that was performed on the upper cervical spine. Such treatment is thought to affect the functioning of the vagus nerve and thereby influence the parasympathetic branch of the autonomic nervous system. The SMT used in the present study was not focused on the upper cervical spine. Nevertheless, our study did show that SMT contributed to the observed shift in the sympathovagal balance towards parasympathetic cardiovascular control. Moreover, according to several studies^.^ [43–45], the cardio-protective effect of physical activity can be partly explained by the increase in parasympathetic activity. Accordingly, SMT also increased the parasympathetic cardiac control. Therefore, an HRV, SBP variability and baroreflex analysis provides a useful tool for clinicians to quantify and objectify the beneficial effects of SMT.

#### The perceived level of pain (NPS)

Although there was no difference between groups of the treatment on NPS, the NPS decreased with both SMT and Sham treatment. The score decreased over time during the first session of treatment and also during the second treatment session, indicating that this was due to the placebo effect. The decrease in NPS during the experiment can be explained by both the experimental period and the treatment. However, the increase of the vagal components of cardiovascular variability with SMT treatment can only partly be explained by the decrease in NPS during treatment sessions.

Although the clinical use of the NPS score has been validated^.^ [34, 46], it remains a subjective estimation of the level of pain; thus, it does not provide a direct estimation of the total effectiveness of treatment. In the present study, the NPS score did not allow us to differentiate between the outcomes of SMT and the Sham treatment, whereas the cardiovascular variability and vagal components (RMSSD, HF-RR and HF-BRS) increased in the SMT group and seem to be more sensitive than the NPS score for detecting the level of the SMT effectiveness. So the analysis of the cardiovascular autonomic control seems to be a valid tool for estimating, at least indirectly, the level of pain [24] and SMT effectiveness.

#### Methodological limitations

In our study, we used both manipulation and mobilization in the SMT group and the subjects received the same treatment. We were confident in using both these types of manual therapy, as studies [47, 48] have shown that there is no real difference in effect with manipulation or mobilization.

The main results of this study were that the vagal modulation of heart rate (RMSSD, HF-RR and HF- baroreflex sensitivity) was significantly higher after SMT than after the Sham intervention (Fig. 2). It is interesting that proponents of SMT believe it has a clinical effect yet are so worried that the mere light touch could increase the sympathetic nervous activity. However, we could not find a credible reference about the possible effect of Sham intervention on the sympathetic nervous system. Yet, we found in this study that neither LF SBP nor LF BRS differed between Sham and SMT.

The NPS score decreased over time during the first and the second session of treatment. However, we did not find a significant difference between the two sessions of treatment (D1 vs. D7). Perhaps this can be explained by the fact that two sessions of treatment are insufficient to have a significant improvement in the physiological components.

Moreover, other factors such as the psychological stress or age difference might influence the results of this study.

Furthermore, there is no significant effect between SMT and Sham for most of the variables of HRV, SBP and pain. These results may be due to the small sample size. Therefore, it would be important to consider the number of study subjects included in studies of this type. Although, our results are interesting, they should be considered with caution and whether these results are generalizable to other types of back pain populations, or indeed to people in general, is not known.

#### Perspectives

This study has been conducted with patients suffering from acute back pain. It has already been shown that the presence of chronic pain significantly alters the nature of the interactions between blood pressure, baroreflex sensitivity and the perceived level of pain^.^ [24]. Thus, it should be of interest to undertake a similar study to quantify the effect of SMT in patients with chronic back pain using a similar cardiovascular variability analysis, as this patient group is more likely to suffer the ill effects of a pain-induced imbalance of the autonomic nervous system, and consequently, might have more secondary benefits from spinal manipulative therapy.

## Conclusion

Our results show that SMT shifted the sympathovagal balance towards an increase in the parasympathetic cardiovascular autonomic control in patients suffering from acute back pain. This effect is known to be beneficial to health^.^ [10]. Whereas the numeric pain scale (NPS) measure did not provide any information about the difference of the perceived level of pain after SMT and Sham. Therefore, this cardiovascular analysis (HRV, SBP variability and baroreflex) shows promise as a useful tool for clinicians to quantify and objectively assess the beneficial effects of SMT. However, these results should be confirmed on a larger number of patients of both sexes with different pathologies.

## Abbreviation

BP: Blood pressure
D: Day
ECG: Electrocardiogram
*f*HF: Breathing frequency
HF: High frequency power
HRV: Heart rate variability
HVLA: High-velocity, low-amplitude
LF: Low frequency power
NPS: Numeric pain scale
PRE: Pretreatment
RMSSD: The root mean square differences of successive RR intervals
RR: Beat to beat intervals
SBP: Systolic blood pressure
Sham: Sham treatment
SMT: Spinal manipulative treatment
SPWVD: Smoothed pseudo Wigner-Ville distribution
